# Two Cases of Metastatic Castration‐Resistant Prostate Cancer With Long‐Term Survival Treated With Abiraterone

**DOI:** 10.1002/ccr3.71617

**Published:** 2025-12-03

**Authors:** Minami Une, Shinya Yamamoto, Honoka Fuse, Ryo Fujiwara, Junji Yonese, Kenjiro Noda

**Affiliations:** ^1^ Department of Urology Nishitokyo Chuo General Hospital Tokyo Japan; ^2^ Department of Genitourinary Oncology Cancer Institute Hospital Japanese Foundation for Cancer Research Tokyo Japan

**Keywords:** abiraterone, androgen receptor pathway inhibitor, complete response, long‐term survival, metastatic castration‐resistant prostate cancer

## Abstract

We report two cases of metastatic castration‐resistant prostate cancer (mCRPC) with long‐term survival following treatment with abiraterone. Despite the generally temporary efficacy of abiraterone, both patients remained progression‐free for over 5 years, suggesting that some mCRPC patients may achieve durable responses with abiraterone.

**Abbreviations**
ADT: androgen deprivation therapy
ARPIs: androgen receptor pathway inhibitors
CAB: combined androgen blockade
CT: computed tomography
GS: Gleason score
mCRPC: metastatic castration‐resistant prostate cancer
mCSPC: metastatic castration‐sensitive prostate cancer
PC: prostate cancer
PSA: prostate‐specific antigen
r‐PFS: radiographic progression‐free survival
VMAT: volumetric modulated arc therapy

## 1 Introduction

It is well known that the prognosis for PC is generally more favorable compared with that for other cancers. However, once the disease progresses to mCRPC, the prognosis significantly worsens, with an average survival of approximately 1.6 years [1]. In this case the first treatment for mCSPC was ADT monotherapy (±non‐steroidal antiandrogen). ARPI treatment is essential for patients who progress to mCRPC from mCSPC; however, the effect of ARPIs on mCRPC is generally temporary [2, 3]. In a randomized phase III COU‐AA‐302 trial, the median r‐PFS of mCRPC patients treated with abiraterone was 16.5 months [4]. Meanwhile, the rate of r‐PFS at 12 months was 65% among patients treated with enzalutamide [3]. To our knowledge, there have been no previous reports of mCRPC in which the therapeutic effects of ARPIs have lasted for a long time. We present two representative cases of long‐term survivors with CRPC treated with abiraterone. These two cases were selected because their clinical courses have been thoroughly observed by the author as the attending physician.

## 2 Case History/Examination

### 2.1 Case Report 1

**FIGURE 1:**
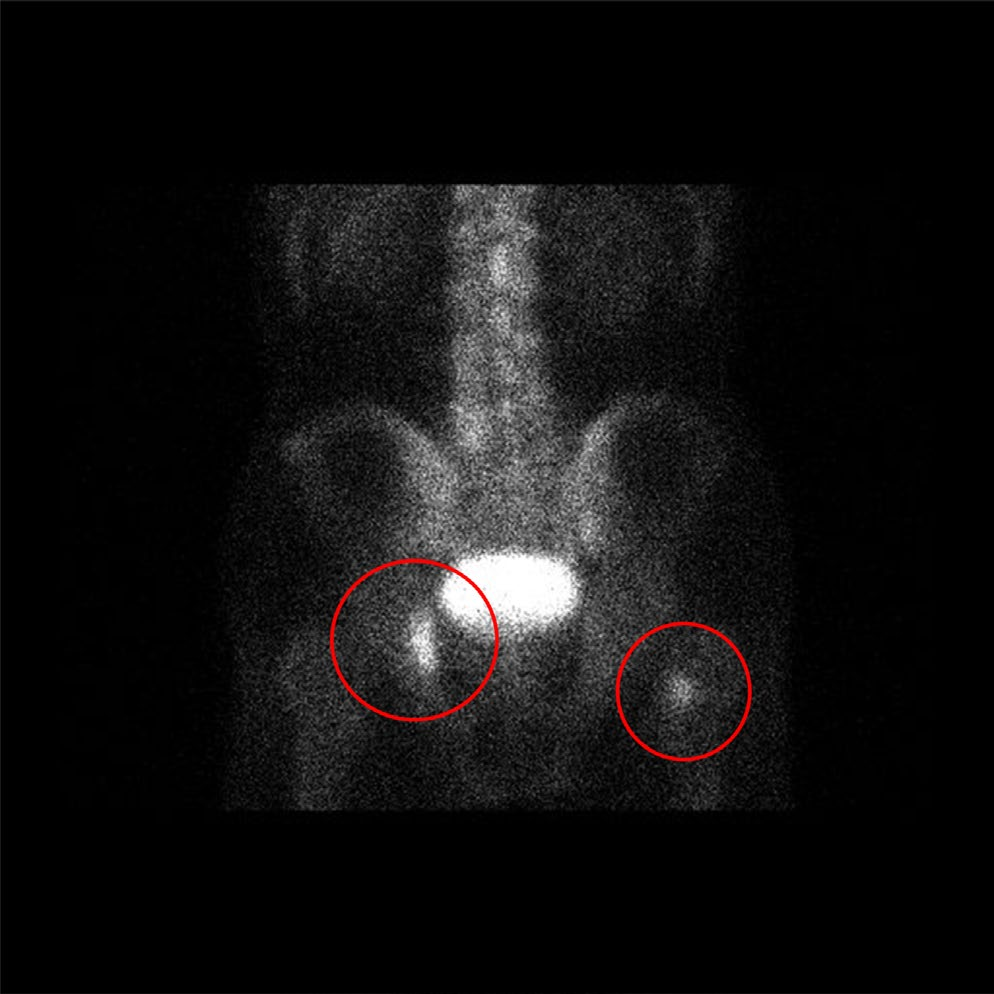
Left ischium and right femoral trochanteric bone metastases at the time of prostate cancer diagnosis.

**FIGURE 2:**
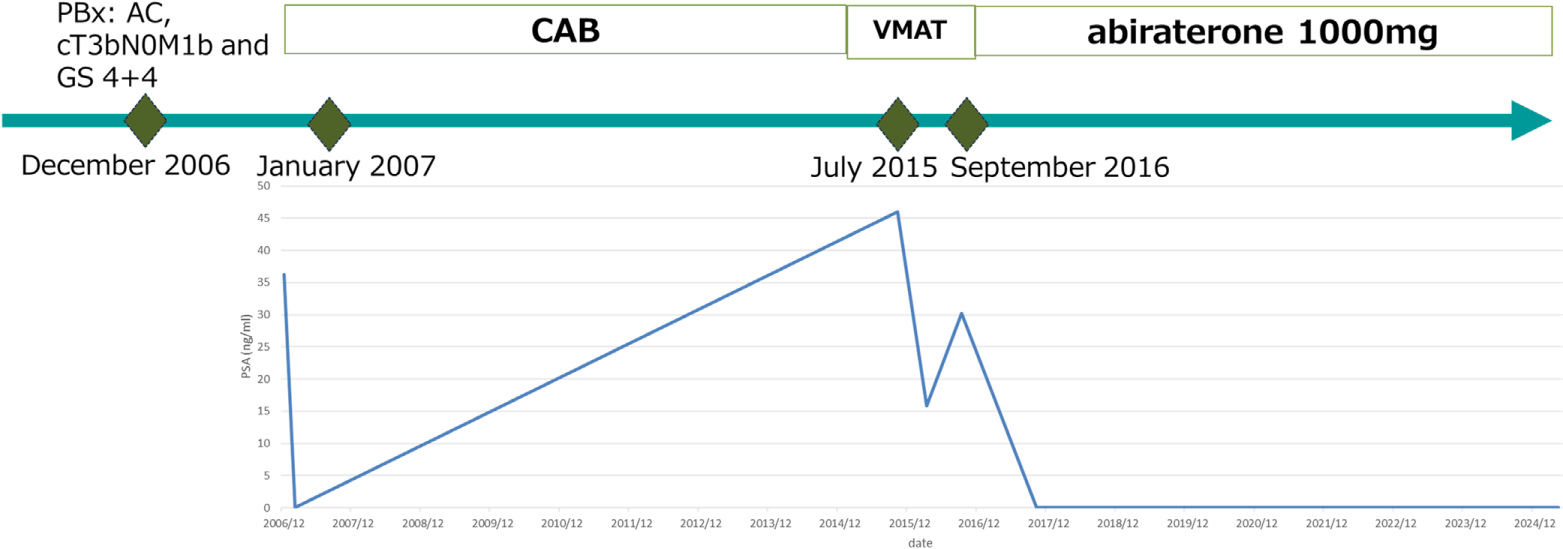
Change in serum PSA concentration in a patient treated with CAB, VMAT, and abiraterone. AC, adenocarcinoma; GS, Gleason score; PBx, prostate biopsy.

A 76‐year‐old male with a PSA level of 36.2 ng/mL was referred to our hospital in December 2006. He was diagnosed with prostatic adenocarcinoma cT3bN0M1b and GS 4 + 4 with a transrectal biopsy. Bone imaging revealed left ischium and right femoral trochanteric bone metastases (Figure 1). In January 2007, CAB was initiated, and nadir PSA was 0.02 ng/mL. In September 2014, his PSA level had again elevated to 16.85 ng/mL. Therefore, he was diagnosed as having mCRPC. Choline CT scan and digital rectal examination revealed only local recurrence in the right lobe of the prostate in July 2015. Due to local recurrence, VMAT; 72 gray/36 fraction was indicated for his prostate. His PSA temporarily decreased to 15.86 ng/mL; however, abiraterone 1000 mg plus prednisolone 10 mg oral administration was introduced to him in September 2016 because of his second PSA elevation. His PSA decreased from 30.17 to 0.01 ng/mL quickly and he has been stabilized for 8 years (Figure 2).

### 2.2 Case Report 2

**FIGURE 3:**
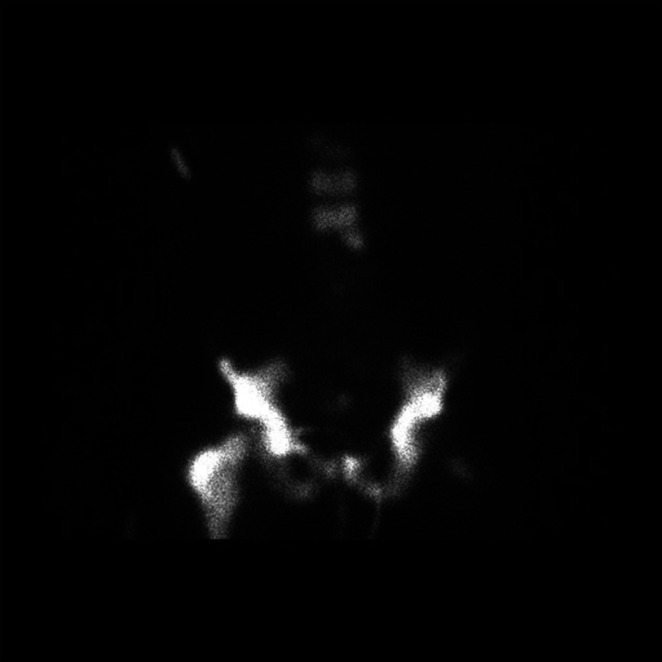
Multiple metastases to vertebrae and pelvic bone at the time of prostate cancer diagnosis.

**FIGURE 4:**
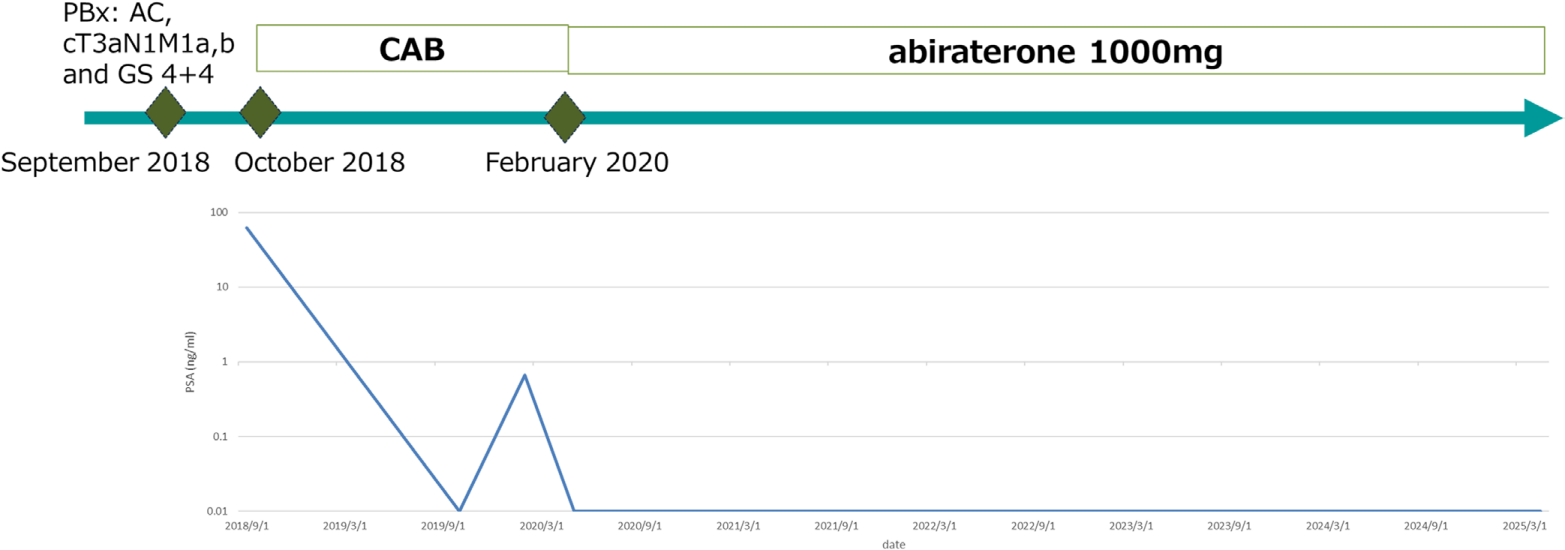
Change in serum PSA concentration in a patient treated with CAB and abiraterone. AC, adenocarcinoma; GS, Gleason score; PBx, prostate biopsy.

An 85‐year‐old male with a PSA level of 62.36 ng/mL was referred to our hospital in September 2018 and diagnosed with prostatic adenocarcinoma cT3aN1M1a, b and GS of 4 + 4 with transperineal biopsy. Bone imaging and CT scan revealed multiple bone and retroperitoneal lymph node metastases, respectively (Figure 3). In October 2018, although CAB therapy was started, his PSA level decreased to 0.01 ng/mL; thereafter, his PSA gradually increased again. In February 2020, because his PSA increased to 0.66 ng/mL, abiraterone 1000 mg plus prednisolone 10 mg oral administration was introduced. Since the introduction of abiraterone plus prednisolone, his PSA has remained stable at 0.01 ng/mL for 5 years (Figure 4).

## 3 Differential Diagnosis

Both patients were diagnosed based on PSA levels, imaging studies, and histological confirmation of prostate adenocarcinoma. Local recurrence of case1 was confirmed by imaging and physical exam.

## 4 Outcome and Follow‐Up

In both cases, long‐term PSA control has been achieved with abiraterone and prednisolone. Case 1 remained recurrence‐free for 8 years, and case 2 for 5 years. Both patients showed no evidence of disease progression and continued to be followed with stable disease status.

## 5 Discussion

These two mCRPC cases, whose therapeutic effects of abiraterone have lasted for a long time, are exceedingly rare. Furthermore, in one of the two cases, no tumor progression was observed for 8 years despite discontinuation of treatment. Accordingly, these mCRPC patients might have achieved long‐term disease control, possibly approaching complete remission with abiraterone. In our institution, only six (1.5%) out of 399 patients with mCRPC achieved long‐term survival with ARPIs, indicating that the probability of such outcomes is extremely low. This finding suggests that some mCRPC patients may have the potential of attaining long‐term survival using ARPIs, which are generally temporary in effect. Here, this finding has raised two suggestions: (1) what kind of mCRPC can achieve long‐term survival with ARPI? And (2) of the several types of ARPI, which ARPI can be used to achieve long‐term survival in mCRPC? As both suggestions are currently speculative, future research will be needed. However, because previous reports have indicated that most low‐volume mCSPC patients experience long‐term survival [5], and mCSPC patients with long‐term effects following first ADT have a good prognosis [6], the long‐term effects in patients with mCRPC treated with ARPIs may also be applicable to the findings in these reports. These two cases were low‐volume mCSPC at first diagnosis, and the therapeutic effect period of the first ADT, in case 1, was 7 years. Additionally, the first case developed mCRPC and underwent VMAT prior to initiating abiraterone. Reports indicate that external‐beam radiotherapy for castration‐resistant prostate cancer has been associated with improved long‐term survival and increased non‐recurrence rates [7]. In this case, since localized recurrence was evident through a digital rectal examination and CT scan, adding radiation therapy might be effective for such cases.

Meanwhile, regarding GS, several studies have reported that GS does not influence the therapeutic efficacy of abiraterone in mCRPC patients [8, 9]. Additionally, several studies have reported that there is no difference in therapeutic effects among ARPIs [10, 11]. Abiraterone was used in the present cases. However, there has been one report of mCRPC patients with long‐term efficacy treated with enzalutamide [12]. A point similar to this case is the long period before progression to mCRPC. As mentioned previously, cases with a long duration of ADT response in the CSPC study tend to show significant efficacy regardless of the type of ARPI used. A key difference is that the patient in this case report underwent curative treatment for localized prostate cancer at the first visit. Whether curative treatment prolongs survival by ARPI remains unclear. A limitation of this case report is that predictive factors for long‐term survival in mCRPC remain unclear. Furthermore, although genetic testing was not performed in any of these cases, genetic factors may have also influenced treatment response. SPOP mutations affect the AR protein and transcription programs, and several cohort analyses have reported favorable outcomes with ARPI therapy [13, 14]. It is possible that SPOP gene mutations were also present in this case. Therefore, in the near future, some patients with mCSPC treated with ADT plus ARPI may acquire long‐term survival.

## 6 Conclusion

Although the percentage is low, some mCRPC patients can gain long‐term survival if treated with abiraterone.

## Author Contributions

**Minami Une:** project administration, writing – original draft. **Shinya Yamamoto:** project administration, writing – review and editing. **Honoka Fuse:** writing – review and editing. **Ryo Fujiwara:** writing – review and editing. **Junji Yonese:** writing – review and editing. **Kenjiro Noda:** writing – review and editing.

## Funding

This work was supported by the Hubei Provincial Department of Education Scientific Research Plan for Young Talent Project (Q20234306) and the Doctoral Start‐up Funding of Jingchu University of Technology (YY202415).

## Ethics Statement

The authors have nothing to report.

## Consent

Written informed consent for publication of this case report and accompanying images was obtained from the patients.

## Conflicts of Interest

The authors declare no conflicts of interest.

## Data Availability

The data that support the findings of this study are available on request from the corresponding author. The data are not publicly available due to privacy or ethical restrictions.
